# Treatment of Alzheimer’s disease in Brazil: I. Cognitive disorders

**DOI:** 10.1590/S1980-57642011DN05030005

**Published:** 2011

**Authors:** Francisco de Assis Carvalho do Vale, Ylmar Corrêa Neto, Paulo Henrique Ferreira Bertolucci, João Carlos Barbosa Machado, Delson José da Silva, Nasser Allam, Márcio Luiz Figueredo Balthazar, Group Recommendations in Alzheimer’s Disease and Vascular Dementia of the Brazilian Academy of Neurology

**Affiliations:** 1 Federal University of São Carlos (UFSCar), Department of Medicine (DMed), São Carlos SP, Brazil; 2 Federal University of Santa Catarina (UFSC), Department of Internal Medicine, Florianópolis SC, Brazil; 3 Federal University of São Paulo (UNIFESP), Sector of Behavioral Neurology - Escola Paulista de Medicina, São Paulo SP, Brazil; 4 Aurus IEPE – Institute of Research and Education on Aging of Belo Horizonte; Faculty of Medical Sciences of Minas Gerais (FCMMG), Department of Geriatric Medicine of Hospital Mater Dei, Belo Horizonte MG, Brazil; 5 Neurosciences Center of Hospital das Clinicas of the Federal University of Goiás (UFG). Integrated Institute of Neurosciences (IINEURO), Goiânia GO, Brazil; 6 University of Brasilia (UnB), Laboratory of Neurosciences and Behavior, Brasília DF, Brazil; 7 University of Campinas (UNICAMP), Faculty of Medical Sciences (FCM), Department of Neurology, Campinas SP, Brazil

**Keywords:** Alzheimer’s disease, dementia, cognitive disorders, treatment

## Abstract

This article reports the recommendations of the Scientific Department of Cognitive Neurology and Aging of the Brazilian Academy of Neurology for the treatment of Alzheimer’s disease (AD) in Brazil, with special focus on cognitive disorders. It constitutes a revision and broadening of the 2005 guidelines based on a consensus involving researchers (physicians and non-physicians) in the field. The authors carried out a search of articles published since 2005 on the MEDLINE, LILACS and Cochrane Library databases. The search criteria were pharmacological and non-pharmacological treatment of cognitive disorders in AD. Studies retrieved were categorized into four classes, and evidence into four levels, based on the 2008 recommendations of the American Academy of Neurology. The recommendations on therapy are pertinent to the dementia phase of AD. Recommendations are proposed for the treatment of cognitive disorders encompassing both pharmacological (including acetyl-cholinesterase inhibitors, memantine and other drugs and substances) and non-pharmacological (including cognitive rehabilitation, physical activity, occupational therapy, and music therapy) approaches. Recommendations for the treatment of behavioral and psychological symptoms of dementia due to Alzheimer’s disease are included in a separate article of this edition.

## Abstract (pt)

Esse texto apresenta as recomendações da Academia Brasileira de Neurologia, por intermédio do seu Departamento Científico de Neurologia Cognitiva e do Envelhecimento, para o tratamento da doença de Alzheimer (DA) no Brasil, enfocando os transtornos cognitivos. Trata-se de uma revisão ampliada das diretrizes publicadas em 2005, resultada de um consenso envolvendo pesquisadores da área, médicos e não médicos. Os autores realizaram uma busca de artigos publicados a partir de 2005 nas bases MEDLINE, LILACS e Cochrane Library. A busca foi direcionada para tratamento farmacológico e não farmacológico dos transtornos cognitivos da DA. Os estudos foram categorizados em quatro classes e as evidências em quatro níveis, com base nas recomendações da Academia Americana de Neurologia publicadas em 2008. As recomendações terapêuticas referem-se à fase demencial da DA. Apresentam-se recomendações para o tratamento dos transtornos cognitivos, tanto farmacológico (incluindo inibidores da acetilcolinesterase, memantina e outros fármacos e substâncias), como não farmacológico (incluindo reabilitação cognitiva, atividade física, terapia ocupacional e musicoterapia). As recomendações para o tratamento dos sintomas comportamentais e psicológicos da demência da DA são apresentadas em outro artigo desse fascículo.

## Introduction

In 2005, the Scientific Department of Cognitive Neurology and Aging (DCNCE-ABN) of the Brazilian Academy of Neurology published a set of recommendations and suggestions for the treatment of Alzheimer’s disease (AD).^1^ The present report comprises an updated version of these recommendations for treatment of cognitive disorders based on current literature. The recommendations are part of a consensus effort, involving a multi-disciplinary group of specialist researchers (physicians and non-physicians) which is overseen by the DCNCE-ABN. Recommendations for the treatment of behavioral and psychological symptoms of Alzheimer’s disease dementia are included in a separate article of this edition.

The authors carried out a search of articles published since 2005 on the MEDLINE (PubMed), LILACS and Cochrane Library databases. The theme was split into two topics for the search:

(I) pharmacological treatment, including acetyl-cholinesterase inhibitors, memantine and other drugs (*Ginkgo biloba* extract, selegiline, Vitamin E, non-steroidal anti-inflammatory drugs, statins, estrogens, omega 3, Vitamins B and folic acid); and
(II) non-pharmacological treatment including rehabilitation/practice/cognitive training, physical activity, occupational therapy, music therapy, physiotherapy and speech therapy.

Studies retrieved were categorized into four classes, and evidence into four levels (Table 1), based on the 2008 recommendations by the American Academy of Neurology.^2,3^ A draft of the recommendations was then presented to a panel of researchers from various disciplines (Neurology, Psychiatry, Geriatrics, Neuropsychology and Speech therapy) for discussion and consensus.

**Table 1:** Level of evidence.

**A.**	Established as effective, ineffective or prejudicial (or establish as useful/predictive or not useful/predictive) for a given condition in the specified population. (Classification level A requires at least two consistent Class I studies)*.
**B.**	Probably effective, ineffective, or prejudicial (and probably useful/predictive or not useful/predictive) for a given condition in the specified population. (Classification level B requires at least one consistent Class I or two Class II studies).
**C.**	Possibly effective, ineffective, or prejudicial (and probably useful/predictive or not useful/predictive) for a given condition in the specified population. (Classification level C requires at least one consistent Class II, or two Class III studies).
**U.**	Insufficient or conflicting data; based on current knowledge, the treatment (trial, prediction) is not proven.

* In exceptional cases, a convincing Class I study may suffice for A recommendation if: (1) all criteria are fulfilled, (2) the magnitude of the effect is large (relative degree of better result >5 and lower limit of confidence interval >2).

In April 2011, a work group from the American National Institute on Aging and the Alzheimer’s Association published recommendations for the diagnosis of dementia due to Alzheimer’s disease^4^ consisting of a revision of the diagnostic criteria for AD published in 1984.^5^ In the same period, the group also published recommendations the diagnosis of mild cognitive impairment due to AD^6^ along with recommendations for application in the research setting containing criteria for the so-called “pre-clinical” stages of AD.^7^ The recommendations for treating AD proposed by the ABN apply to the dementia phase of the disease, whilst the present studies assessed were based on the definition of probable AD from the 1984 criteria.

This report is organized under two sections (pharmacological treatment and non-pharmacological treatment). With regard to the recommendations related to pharmacotherapy, it should be noted that these are based on scientific studies, whereas the prescribing physician must still check whether the drug is approved by the National Health Surveillance Agency (ANVISA).

## Pharmacological therapies

### Acetyl-cholinesterase inhibitors (AChEI)

In AD, changes occur at different points in the cholinergic pathways. Relatively early in the disease course, compromise and neuronal loss in the *nucleus basalis of* Meynert occurs, leading to loss of choline acetyl-transferase (ChAT) with consequent reduced ability to synthesize acetylcholine (ACh). Additionally, in early phases of AD, there is also loss of nicotinic receptors.^8^ This ultimately results in a fall in cholinergic activity. Given the experimental evidence outlined above, it is reasonable to assume that at least some of the symptoms of AD stem from this deficit, and that remedying it could therefore lead to clinical improvements in AD patients.

One possible approach to manage this deficit is by inhibiting the degradation of ACh such that the lower quantity of neurotransmitter produced is used more effectively. Attempts to achieve this have been made over the past three decades by use of physostigmine. Improvement in memory was observed but its clinical use became unviable for two reasons:

(i) its short half-life meant frequent administration and;
(ii) its peripheral action leads to collateral effects such as nausea, vomiting and abdominal pain. Later investigation showed that another inhibitor of cholinesterase, tetrahydroaminoacridine (tacrine), was able to improve the condition of AD patients.^10^

Approved by the regulatory agencies, this was the first drug to be used on a large scale to treat AD. Nevertheless, despite its advantages over physostigmine, tacrine requires four daily applications and causes hepatic alterations in 30 to 40% of patients. The drug fell into disuse upon the advent of new AChEIs. Besides tacrine, other drugs approved in Brazil for the treatment of mild-to-moderate AD include rivastigmine, donepezil and galantamine.

Rivastigmine is a carbamate which irreversibly inhibits AChE. The drug also inhibits butyrylcholinesterase although the clinical relevance of this action is unclear. It has a short half-life of around one hour but inhibition of the enzyme persists for 10 to 12 hours. The drug’s short half-life requires it to be administered twice a day, in the morning and evening in oral dosage form. More recently, a slow-release transdermal patch was launched requiring daily administration. The majority of the drug is metabolized by AChE and renally excreted. Large-scale, double-blind, placebo-controlled trials have shown greater efficacy of rivastigmine over placebo.^11^

Donepezil is a piperidine essentially metabolized by the liver, with a long half–life of around 70 hours, allowing administration in a single night-time dose. Large-scale, double-blind, placebo-controlled trials have shown greater efficacy of donepezil compared to placebo.^12^

Galantamine is a phenanthrene with a plasma half-life of about 7 hours which is partially metabolized by the liver and partly excreted directly by the kidneys. A difference between galantamine and other AChEIs is that the drug has a modulating action on nicotinic receptors, although the clinical relevance of this remains obscure. Double-blind, placebo-controlled trials have shown the drug to be superior over placebo.^13^

Theoretically, the expected response for an AChE inhibitor should be an initial improvement in symptoms, which then wanes with AD progression. However, evidence suggests these drugs can partially stabilize this progression thereby slowing disease evolution. Overall, the effects are modest but significant, showing improvements in cognition, behavior and functionality. Few studies have been specifically designed to compare differences among the inhibitors available, and results so far have been either conflicting or showed no difference among the three drugs cited.^14^ Similarly, the benefits of inhibitors over one another in terms of side effects is also unclear. Comparison of different studies, notwithstanding all the limitations inherent to this method of analysis, appear to show slightly greater tolerability for donepezil in terms of gastrointestinal collateral effects (nausea, vomiting and diarrhea).^15^ No comparative studies involving transdermal rivastigmine are available although the patch form is associated with fewer side effects than the oral capsule.^16^ Several general principles should be born in mind concerning AChEIs: always start at the minimum dose, with dosage escalation preferably at 4-week intervals, keeping the dose stable for a minimum of 2 months in order to assess patient response. Initial and maintenance doses are given in Table 2. Response is generally modest while a significant proportion of patients having no response. Initial response can be lost and in this case it is possible to try switching over to another AChEI since loss of response to one drug does not necessarily imply this will also be the case for the others.

**Table 2:** Posology of cholinesterase inhibitors.

Drug	Route of administration	Initial daily dosage	Daily maintenance dosage	Doses per day
Donepezil	Oral	5 mg	5-10 mg	One
Galantamine	Oral	8 mg	16-24 mg	One
Rivastigmine	Oral Transdermal*	3 mg 4.6 mg	6-12 mg 9.5 mg	Two One

* Level of evidence B.

**Recommendations** - The use of cholinesterase inhibitors is effective for mild-to-moderate Alzheimer’s disease (Level of evidence A).

#### AChEI IN SEVERE AD

The studies which led to the approval of AChEI for use in AD typically included individuals with mild to moderate AD. In principle, with increased loss of cholinergic neurons, the chances of response are lessened but some effect cannot be ruled out. A controlled study with galantamine in institutionalized elderly with severe AD (MMSE score of between 5 and 12) showed a favorable difference of the drug on cognitive assessment, but not for activities of daily living.^17^ A retrospective analysis of the effect of rivastigmine transdermal patch or capsule in a controlled study showed that individuals with severe AD (score from 7 to 12 on MMSE) had a significantly better response compared to placebo for cognition, activities of daily living and global clinical impression.^18^ Three controlled studies with donepezil in severe AD showed a significantly better result in the treated group versus placebo for cognition, activities of daily living, and global assessment.^19^ Donepezil is approved for management of moderate to moderately severe AD. For the other two AChEIs, prescription for this stage, despite the evidence from clinical trials, is off-label in Brazil since this indication has yet to gain approval from the regulatory agencies.

**Recommendations** - The use of cholinesterase inhibitors is effective in severe AD (Level A).

### Memantine

Memantine is a non-competitive antagonist with moderate affinity for NMDA (N-methyl-d-aspartate) type receptors of glutamate and this promotes a reduction in the pathologic neuronal excitotoxicity induced by this neurotransmitter and mediated by calcium. Also, it may possibly facilitate neurotransmission and neuroplasticity. Oral absorption is complete and half-life long (60-80h). It has a moderate bond to plasma protein (45%). Metabolization is minimal in the CYP450 system and excretion is renal (57-82% unaltered). Recommended posology is an initial dose of 5 mg/day, escalated to 20 mg/day (Table 3). Since its elimination is renal and it hardly uses the system of the P 450 hepatic cytochrome, it exhibits little interaction with other drugs. Moreover, it appears to have no influence on the metabolism of AChEIs. It has good tolerability with the most frequent adverse effects being agitation, diarrhea, insomnia, disorientation, hallucinations, dizziness, cephalea, fatigue, anxiety, hypertonia and vomiting.^20^ The drug was approved in Brazil for use in patients with moderate to advanced Alzheimer’s in 2004.

**Table 3:** Posology of memantine.

Route of administration	Oral
Doses per day	Two (single daily dose during first two weeks)
Initial daily dosage	5 mg
Dosage escalation	Every 1-2 weeks
Max. daily dosage	20 mg
Administration with food	Not necessary

#### MEMANTINE IN MODERATE-TO-SEVERE AD

Two randomised controlled clinical trials were pivotal for the approval of memantine by the regulatory bodies in the United States, some European countries and in Brazil. These studies confirmed the drug’s clinical efficacy, albeit slight, and tolerability of memantine in individuals with moderate-to-severe Alzheimer’s disease, used alone^21^ or in association with donepezil.^22^

A meta-analysis of 6 clinical trials concluded that memantine offered clinical efficacy in terms of cognition, behavior and functionality as well as good tolerability in individuals with moderate to severe AD.^23^

A prospective cohort showed that a combination of AChEI with memantine was more effective in slowing cognitive and functional decline in persons with moderate to severe AD compared to monotherapy with AChEI or no pharmacotherapy.^24^ There is a good rationale for combining memantine with AChEI, since their respective mechanisms of actions are entirely different and memantine appears to have no effect on the metabolism of AChEIs.^20,25,26^

A 6-year prospective cohort study showed that use of memantine alone or associated to AChEI was effective for functionality but not for cognition in moderate to severe Alzheimer’s disease patients.^27^ A recent review concluded that memantine has some degree of efficacy in cognition and functionality, as well as good tolerability in individuals with moderate to severe AD.^28^

**Recommendations** - The use of memantine, alone or associated with AChEI, is effective in individuals with moderate to severe AD (Level A).

#### MEMANTINE IN MILD TO MODERATE AD

A number of clinical trials have been conducted in mild-to-moderate AD patients with memantine alone or associated with an AChEI although results were conflicting.

A randomized placebo-controlled clinical trial in mild to moderate AD patients showed that treatment with memantine alone resulted in significantly better outcomes on measures of cognition, global status and behavior.^29^ Another randomized placebo-controlled clinical trial in individuals with mild to moderate AD using an AChEI (donepezil, galantamine or rivastigmine) revealed that memantine was no better than placebo on measures of cognition, behavior and functionality.^30^

A meta-analysis of 6 clinical trials concluded that memantine had homogenous and significant effects on measures of global assessment and cognition,^31^ but serious methodological flaws were exposed and the results of the study ere contested because two of its authors were found to be employees of the study sponsor.^32,33^

A cohort study concluded that the use of memantine together with AChEI in individuals with mild AD can be detrimental to global cognition.^34^

In conclusion: memantine has shown clinical efficacy, albeit slight, when used alone or in combination with AChEI, in subjects with moderate to severe AD. The data on the clinical efficacy of memantine, used alone or co-administered with AChE, in individuals with mild to moderate AD are highly conflicting; memantine exhibits good tolerability and safety at all stages of AD.

**Recommendations** - The latest evidence from the scientific literature does not support the use of memantine, either alone or associated with AChEI, in the treatment of the initial stages of AD (Level U).

### Other drugs and substances

#### GINKGO BILOBA

The extract of *Ginkgo biloba* EGb 761 contains active ingredients which promote increased blood flow to the brain through vasodilation and reduced blood viscosity, besides reduced free radicals in nervous tissue.^35^ Laboratory models have associated its action with pathological mechanisms of AD such as aggregation and amyloid toxicity, mitochondrial dysfunction, insulin resistance and oxidative injury.^35,36^ The effects of EGb761 in elderly with preserved cognition included objective improvement in cognitive processing speed as well as subjective perception of improved memory.^37^ Nonetheless, based on a recent review of 36 randomized clinical trials, nine of which ran for at least six months (2016 patients), concluded the actual benefits of EGb761 for the treatment of cognitive impairment and dementia of Alzheimer’s type were unclear and inconsistent.^38^ Similarly, the results of some multi-center studies failed to confirm efficacy of EGb for the prevention of cognitive decline or of dementia due to Alzheimer’s disease.^39-41^

#### VITAMIN E (ALPHA-TOCOPHEROL)

Given the evidence that oxidative stress can contribute to the pathogenesis of Alzheimer’s disease dementia, the use of antioxidant measures appears to have role in treatment.^42^ An extensive population-based prospective cohort study showed a lower risk of dementia of the Alzheimer’s type following food intake of Vitamin E.^43^ The benefit of treatment using a high dosage (2000 UI/day) of vitamin E was initially shown^44^ but a later study in individuals with mild cognitive impairment of the amnestic type failed to confirm these positive findings.^45^ However, a recent systematic review concluded that current data on Vitamin E supplementation for the treatment of mild cognitive decline and Alzheimer’s disease dementia are lacking.^46^ Moreover, a broad meta-analysis study showed that several groups (adults, older adults, healthy subjects, individuals with various diseases) undergoing treatment with a range of Vitamin E dosages, exhibited greater risk of all-cause mortality compared to control groups, with negative outcomes associated to higher doses. The study concluded that supplementation with doses higher than 400UI/day should be avoided until fresh evidence of the efficacy of supplementation is available from rigorous clinical trials.^47^ Consequently, a drastic drop in prescribing of vitamin E for the treatment of AD dementia ensued.^48^

#### SELEGILINE (L-DEPRENYL)

Only one study employing an acceptable method evidenced benefits,^44^ although this had a poor risk-benefit ratio. Another comprehensive meta-analysis review found only negligible benefit.^49^

#### OMEGA-3

Epidemiological and laboratory-based studies point to a protective effect of a diet rich in fish and fatty acids, such as docosahexaenoic acid and eicosapentaenoic acid, for AD dementia. Positive effects on weight and appetite were shown in a single study in patients with mild Alzheimer’s disease dementia.^50^ At present, there is no evidence to support the use of Omega-3 supplementation for the prevention of cognitive impairment and dementia or for improving neuropsychiatric symptoms secondary to dementia.^51-53^

#### HOMOCYSTEINE REDUCERS

Elevated blood homocysteine levels occur in AD. Hyperomocysteinemia can contribute to the physiopathology of the disease by vascular mechanisms and direct neurotoxic effects. Even in the absence of vitamin deficiency, homocysteine levels can be reduced by high-dose supplementation of folic acid and vitamins B6 and B12. Preliminary studies with high doses of vitamins however, proved unable to halt cognitive decline in individual with mild-to-moderate dementia of the Alzheimer’s type.^54,55^ A review on the use of folic acid with or without vitamin B12 in healthy elderly and demented people concluded that there is currently no consistent evidence to indicate the two forms of supplementation. Therefore, the results of further long-term studies are needed.^53,56^

#### ESTROGEN

Taken together, the physiological effects of estrogen and epidemiologic data suggest the use of estrogen is potentially beneficial. However, there is insufficient clinical evidence for hormonal replacement therapy at any age to be considered a protective factor for AD dementia. In addition, in view of the known adverse effects, its prescription specifically for treating dementia due to AD cannot currently be justified. Whether age at exposure to hormonal replacement therapy, and the relationship between age at menopause and commencement of treatment, are determinants of risk for AD dementia has yet to be ascertained.^57-62^

#### NON-STEROIDAL ANTI-INFLAMMATORY DRUGS (NSAIDS)

Given the inflammatory reaction involving amyloid plaques in AD, anti-inflammatory drugs may have a potential role in disease treatment. In addition, results of epidemiological studies suggest that anti-inflammatories may exert a neuroprotective affect against AD. A large population-based prospective cohort study found that the prolonged use of NSAIDs can protect against AD.^63^ Moreover, a controlled clinical trial showed that rofecoxib and naproxen do not delay cognitive decline in patients with mild to moderate AD dementia^64^ where the same was found for ibuprofen^65^ and indomethacin.^66^ Randomized clinical studies also reported negative outcomes for use of NSAIDs, and likewise for naproxen and celecoxib, in the prevention of AD dementia.^67^ The profile of collateral effects of NSAIDs, particularly digestive hemorrhaging and cardiovascular risks, associated to use of the drugs has limited their prescription.

#### STATINS

Several basic studies have shown the influence of cholesterol levels on the metabolic pathway of amyloid.^68,69^ However, results of a meta-analysis showed no beneficial effects of treatment by statins for preventing AD dementia.^70^ A 72-week course of 80 mg/day atorvastatin to treat dementia in mild to moderate AD was found to yield no clinically significant benefit.^71^ A recent review including three randomized trials each lasting at least six months, cited a lack of evidence to recommend the use of statins for the treatment of AD dementia.^72^

**Recommendations** - The evidence points to inefficacy of *Ginkgo biloba* extract EGb761, vitamin E, selegiline, Omega-3, homocysteine reducers, estrogen, non-steroidal anti-inflammatory drugs and statins in the treatment of Alzheimer’s disease dementia. Hence, the use of these drugs and substances is not recommended for this purpose (Level A).

## Non-pharmacological therapies

Scientific studies on the non-pharmacological treatment of patients with AD typically have methodological limitations inherent to the difficulties in forming adequate control groups that compare against placebo, besides an absence of examiners blinded to the intervention concerned. Although the number of trials on non-pharmacological treatment for cognitive impairment has risen significantly, there remains a growing need for research in this area to determine the value and cost-benefit ratio of this mode of therapy.

The main premise underlying the practice of cognitive rehabilitation is the ability of the human brain to reorganize after lesions. This ability is retained even in neurodegenerative diseases such as AD, where cognitive compensation mechanisms may come into play. This compensation occurs by activation of intact cortical regions which take on the functions previously carried out by the areas that suffered neurodegeneration.^73^

Different approaches have been tried toward cognitive rehabilitation (including cognitive stimulation, memory rehabilitation, reality orientation therapy, and neuropsychological rehabilitation) in addition to physical activity, music therapy and occupational therapy, among others.

Research shows that cognitive stimulation may prove useful in improving cognition when combined with the use of anticholinesterasic drugs.^74,75^ In a randomised, controlled study with a two-year follow up, the group submitted to a combination of cognitive stimulation and donepezil had less decline and significantly higher MMSE score than the control group during the first year, whereas all groups showed cognitive decline in the second year.^75^

A systematic review showed that training of specific cognitive skills in small groups may also possibly lead to cognitive improvement. Two small randomized controlled studies showed improvements in verbal and visual learning after memory strategies were trained daily or twice per week.^76^

With regard to memory rehabilitation techniques such as reality orientation, a meta-analysis showed a possible positive effect on cognition when class room-based tasks were performed.^77^ In a randomized controlled trial, the effect of reality orientation techniques combined with donepezil use was assessed. An improvement of 2.9 points was observed on the cognitive subscale of the *Alzheimer’s Disease Assessment Scale* (ADAS-COG) compared with patients who used medication alone.^78^ It should be noted that cognitive rehabilitation techniques must be implemented while taking into account individuals’ cultural and psychological characteristics. Some patients, upon becoming aware of their deficits tend to suffer a fall in self-esteem which may lead to depressive symptoms.^79^

Other techniques used in memory training include explicit learning, errorless learning, learning with errors, implicit learning, and external mnemonic cues. To date, few randomized studies proving the efficacy of these techniques have been conducted in large samples. Nevertheless, there are indications that these approaches can be beneficial for cognition when applied in conjunction with anticholinesterasic drugs.^80-82^

Similarly, there are insufficient randomized controlled trials on techniques such as occupational therapy, music therapy and equitherapy to allow their application to be formally indicated for cognitive treatment.

One meta-analysis and two systematic reviews show that individualized physical activity programs are potentially effective for improving functioning in patients with mild to moderate AD.^83-85^ Results of cognitive treatment however, have been modest. Another meta-analysis revealed no cognitive benefit,^86^ whereas a randomized controlled trial showed that a basic physical exercise program (comprising one hour sessions twice weekly) sufficed to delay cognitive and functional decline in AD patients.^87^

**Recommendations** - (1) Cognitive stimulation, reality orientation and specific skills training techniques are possibly effective in the treatment of cognitive disorders in individuals with mild to moderate AD when combined with anticholinesterasic drug use.(Level C); (2) Individualized physical activity programs are potentially beneficial for improving functioning of individuals with mild to moderate AD (Level C), while evidence for efficacy in treating cognitive impairment is lacking (Level U). Despite evidence suggesting these therapeutic approaches yield benefits for AD patients, the current scientific evidence is insufficient to enable definitive conclusions to be drawn.

## GROUP RECOMMENDATIONS IN ALZHEIMER’S DISEASE AND VASCULAR DEMENTIA OF THE BRAZILIAN ACADEMY OF NEUROLOGY

**Amauri B. da Silva** [UNINEURO, Recife (PE)]; **Ana Cláudia Ferraz** [Serviço de Neurologia do Hospital Santa Marcelina (SP)]; **Analuiza Camozzato de Pádua** [Universidade Federal de Ciências da Saúde de Porto Alegre (UFCSPA); Hospital de Clínicas de Porto Alegre (UFRGS) (RS)]; **Antonio Lúcio Teixeira** [Departamento de Clínica Médica, Faculdade de Medicina da Universidade Federal de Minas Gerais, Belo Horizonte (MG)]; **Ayrton Roberto Massaro** [Instituto de Reabilitação Lucy Montoro (SP)]; **Benito Pereira Damasceno** [Departamento de Neurologia da Universidade Estadual de Campinas (SP)]; **Carla Tocquer** [Universidade Federal do Rio de Janeiro (RJ)]; **Carlos Alberto Buchpiguel** [Departamento de Radiologia, Faculdade de Medicina da Universidade de São Paulo (SP)]; **Cássio Machado C. Bottino** [Programa Terceira Idade, Instituto de Psiquiatria do Hospital das Clínicas da Faculdade de Medicina da Universidade de São Paulo (FMUSP) (SP)]; **Charles André** [Faculdade de Medicina - UFRJ; SINAPSE Reabilitação e Neurofisiologia (RJ)]; **Cláudia C. Godinho** [Serviço de Neurologia do Hospital de Clínicas de Porto Alegre, Universidade Federal do Rio Grande do Sul (RS)]; **Cláudia Sellitto Porto** [Grupo de Neurologia Cognitiva e do Comportamento da Faculdade de Medicina da USP (SP)]; **Denise Madeira Moreira** [Departamento de Radiologia Faculdade de Medicina - UFRJ; Setor de Radiologia - INDC - UFRJ (RJ)]; **Eliasz Engelhardt** [Setor de Neurologia Cognitiva e do Comportamento - INDC - CDA/IPUB - UFRJ (RJ)]; **Elza Dias-Tosta** [Presidente da Academia Brasileira de Neurologia, Hospital de Base do Distrito Federal (DF)]; **Emílio Herrera Junior** [Departamento de Medicina Interna, Faculdade de Medicina de Catanduva (SP)]; **Gabriel R. de Freitas** [Instituto D’or de Pesquisa e Ensino; Universidade Federal Fluminense (RJ)]; **Hae Won Lee** [Instituto de Radiologia, Hospital das Clínicas da Faculdade de Medicina da Universidade de São Paulo e Hospital Sírio-Libanês (SP)]; **Ivan Hideyo Okamoto** [Departamento de Neurologia e Neurocirurgia; Instituto da Memória - Universidade Federal de São Paulo - UNIFESP (SP)]; **Jerusa Smid** [Grupo de Neurologia Cognitiva e do Comportamento do Hospital das Clínicas da Faculdade de Medicina da Universidade de São Paulo (FMUSP) (SP)]; **José Antonio Livramento** [Laboratório de Investigação Médica (LIM) 15, Faculdade de Medicina da Universidade de São Paulo (SP)]; **José Luiz de Sá Cavalcanti** [Departamento de Neurologia - INDC - UFRJ; Setor de Neurologia Cognitiva e do Comportamento - INDC - UFRJ (RJ)]; **Letícia Lessa Mansur** [Grupo de Neurologia Cognitiva e do Comportamento do Departamento de Neurologia da FMUSP; Departamento de Fisioterapia, Fonoaudiologia e Terapia Ocupacional da Faculdade de Medicina da USP (SP)]; **Liana Lisboa Fernandez** [Departamento de Ciências Básicas da Saúde, Fundação Universidade Federal de Ciências da Saúde de Porto Alegre (RS)]; **Márcia Lorena Fagundes Chaves** [Serviço de Neurologia do Hospital de Clínicas de Porto Alegre, Universidade Federal do Rio Grande do Sul (RS)]; **Márcia Radanovic** [Laboratório de Neurociências - LIM27, Departamento e Instituto de Psiquiatria da Faculdade de Medicina da Universidade de São Paulo (FMUSP) (SP)]; **Maria Teresa Carthery-Goulart** [Grupo de Neurologia Cognitiva e do Comportamento do Departamento de Neurologia da Faculdade de Medicina da USP; Centro de Matemática, Computação e Cognição, Universidade Federal do ABC (SP)]; **Mônica S. Yassuda** [Grupo de Neurologia Cognitiva e do Comportamento do Departamento de Neurologia da Faculdade de Medicina da USP; Departamento de Gerontologia, Escola de Artes, Ciências e Humanidades da USP (EACH/USP Leste) (SP)]; **Norberto Anízio Ferreira Frota** [Universidade de Fortaleza (UNIFOR), Serviço de Neurologia do Hospital Geral de Fortaleza (HGF) (CE)]; **Orestes Forlenza** [Laboratório de Neurociências - LIM27, Departamento e Instituto de Psiquiatria da Faculdade de Medicina da Universidade de São Paulo (FMUSP) (SP)]; **Paulo Caramelli** [Departamento de Clínica Médica, Faculdade de Medicina da Universidade Federal de Minas Gerais, Belo Horizonte (MG)]; **Regina Miksian Magaldi** [Serviço de Geriatria do Hospital das Clínicas da FMUSP, Centro de Referência em Distúrbios Cognitivos (CEREDIC) da FMUSP (SP)]; **Renata Areza-Fegyveres** [Grupo de Neurologia Cognitiva e do Comportamento do Hospital das Clínicas da Faculdade de Medicina da Universidade de São Paulo (FMUSP) (SP)]; **Renato Anghinah** [Grupo de Neurologia Cognitiva e do Comportamento do Hospital das Clínicas da Faculdade de Medicina da Universidade de São Paulo (FMUSP); Centro de Referência em Distúrbios Cognitivos (CEREDIC) da FMUSP (SP)]; **Ricardo Nitrini** [Grupo de Neurologia Cognitiva e do Comportamento do Hospital das Clínicas da Faculdade de Medicina da Universidade de São Paulo (FMUSP); Centro de Referência em Distúrbios Cognitivos (CEREDIC) da FMUSP (SP)]; **Rodrigo Rizek Schultz** [Setor de Neurologia do Comportamento do Departamento de Neurologia e Neurocirurgia da Universidade Federal de São Paulo, Núcleo de Envelhecimento Cerebral (NUDEC) - Instituto da Memória (UNIFESP) (SP)]; **Rogério Beato** [Grupo de Pesquisa em Neurologia Cognitiva e do Comportamento, Departamento de Medicina Interna, Faculdade de Medicina, UFMG (MG)]; **Sonia Maria Dozzi Brucki** [Grupo de Neurologia Cognitiva e do Comportamento da Faculdade de Medicina da Universidade de São Paulo; Centro de Referência em Distúrbios Cognitivos (CEREDIC) da FMUSP; Hospital Santa Marcelina (SP)]; **Tânia Novaretti** [Faculdade de Filosofia e Ciências, Campus de Marília, da Universidade Estadual Paulista (UNESP) (SP)]; **Valéria Santoro Bahia** [Grupo de Neurologia Cognitiva e do Comportamento do Hospital das Clínicas da Faculdade de Medicina da Universidade de São Paulo (FMUSP) (SP)];
